# Long-term impact of four different strategies for delivering an on-line curriculum about herbs and other dietary supplements

**DOI:** 10.1186/1472-6920-6-39

**Published:** 2006-08-07

**Authors:** Tiffany Beal, Kathi J Kemper, Paula Gardiner, Charles Woods

**Affiliations:** 1 Physican Assistant Program at Wake Forest University School of Medicine. Winston-Salem, NC, 27157, USA; 2 Departments of Pediatrics and Public Health Sciences, Wake Forest University School of Medicine. Winston-Salem, 27157, NC, USA; 3 Division for Research and Education in Complementary and Integrative Medical Therapies, Harvard Medical School, Osher Institute, Boston, MA, 02115, USA

## Abstract

**Background:**

Previous research has shown that internet education can lead to short-term improvements in clinicians' knowledge, confidence and communication practices. We wished to better understand the duration of these improvements and whether different curriculum delivery strategies differed in affecting these improvements.

**Methods:**

As previously described, we conducted a randomized control trial comparing four different strategies for delivering an e-curriculum about herbs and other dietary supplements (HDS) to clinicians. The four strategies were delivering the curriculum by: a) email over 10 weeks; b) email within one week; c) web-site over 10 weeks; d) web-site within one week. Participants were surveyed at baseline, immediately after the course and 6–10 months after completing the course (long-term). Long-term outcomes focused on clinicians' knowledge, confidence and communication practices.

**Results:**

Of the 780 clinicians who completed the course, 385 (49%) completed the long-term survey. Completers and non-completers of the long-term survey had similar demographics and professional characteristics at baseline. There were statistically significant improvements from baseline to long-term follow-up in knowledge, confidence and communication practices; these improvements did not differ by curriculum delivery strategy. Knowledge scores improved from 67.7 ± 10.3 at baseline to 78.8 ± 12.3 at long-term follow-up (P < 0.001). Confidence scores improved from 53.7 ± 17.8 at baseline to 66.9 ± 12.0 at long term follow-up (P < 0.001); communication scores improved from 2.6 ± 1.9 at baseline to 3.6 ± 2.1 (P < 0.001) at long-term follow-up.

**Conclusion:**

This e- curriculum led to significant and sustained improvements in clinicians' expertise about HDS regardless of the delivery strategy. Future studies should compare the impact of required vs. elective courses and self-reported vs. objective measures of behavior change.

## Background

Herbs and dietary supplements (HDS) are the most commonly used complementary medical therapies purchased in the United States [1], leading to concerns about HDS safety and efficacy[2]. Health care professionals have expressed a strong interest in HDS training courses[3-5]. However, face-to-face Continuing Medical Education (CME) courses often fail to result in sustained changes in physician behaviors [6,7]. On the other hand, online CME training has shown improved behavior and knowledge [8].

We previously reported the short-term outcomes of our randomized controlled trial (RCT) comparing four different strategies of delivering an on-line course about HDS to diverse clinicians [9]. The short-term results suggested that all four strategies of the e-curriculum similarly and significantly improved clinicians' knowledge, confidence, and communication practices.

To answer questions about the duration of these improvements and whether any differences between delivery strategies would emerge over a longer follow-up, we prospectively followed up study participants from the earlier RCT six to ten months after they'd completed the initial study.

## Methods

We conducted a prospective 6 to 10 month follow-up of an RCT comparing four different strategies for delivering an e-curriculum about herbs and dietary supplements to diverse health professionals [9]. Baseline surveys questions regarding demographics, professional characteristics, knowledge, confidence and communication scales have been reported previously [9,10]. Dieticians, nurses, pharmacists, physicians, physician assistants, and trainees in one of these health professions were eligible for the study.

The intervention and delivery strategies have been described previously [9,10]. Briefly, the curriculum consisted of 40 case-based self-instructional modules, each of which contained links to evidence-based on-line HDS resources. Enrollees were randomized to one of four different curriculum delivery groups: email delivery over ten weeks (push-drip), email delivery over four days (push-bolus), web availability over ten weeks (pull-drip), and web availability over four days (pull-bolus). The curriculum was delivered in fall, 2004 (concluding in 12/04) and in spring, 2005 (concluding in 4/05). Immediate outcomes were assessed 11 – 15 weeks after randomization.

During the second week of October 2005 (approximately ten months after the first group and six months after the second group had completed the course) all original enrollees were asked to complete a final course evaluation. The email request contained a link to a web page which included the exact same questions as the immediate outcome survey to assess long-term retention and maintenance of knowledge, confidence, and communication practices among course enrollees. Non-respondents received up to three email requests to complete the survey before the November 30, 2005 deadline.

The primary study outcomes have also been described previously [9,10]. Briefly, *knowledge scores *were the percent of the knowledge questions answered correctly (potential range 0, 100%). A *confidence scale *score with a possible range of 19 to 95 was derived from responses to 19 Likert-type questions such as "I feel confident responding to patients' questions about HDS;" it had a Cronbach alpha reliability statistic of 0.96. Respondents who had seen patients within the past 30 days completed the *communications practices scale*, with a range of scores from 0 to 10; the Cronbach alpha reliability statistic was 0.84 for baseline and 0.92 for the immediate outcome assessments for this scale.

Chi-square methods were used for evaluation of associations of categorical variables. For continuous outcomes measures, t tests or analysis of variance (ANOVA) were utilized for normally distributed data, and Mann-Whitney U tests or Kruskal-Wallis tests for non-normally distributed variables. For repeated measures outcomes, paired samples t-tests or Wilcoxon signed rank tests were used, depending on data characteristics. Analyses were performed using SPSS 14.0 (SPSS Inc., Chicago, IL).

This study was approved as "exempt" as an educational research project by the Wake Forest University School of Medicine Institutional Review Board.

## Results

Of the 780 participants who completed the course, 385 (49%) completed the long-term follow-up survey six to ten months later (Table 1). Completers (n = 385) and non-completers (n = 395) of the long-term survey had similar age, gender, and practice characteristics and used a similar number of HDS in the week prior to the baseline survey (average of 5.6). There were no significant differences between the completers and non-completers by curriculum delivery strategy, baseline confidence, or communication scores. Knowledge scores were, on average, 1.5% higher among completers.

**Figure 1:**
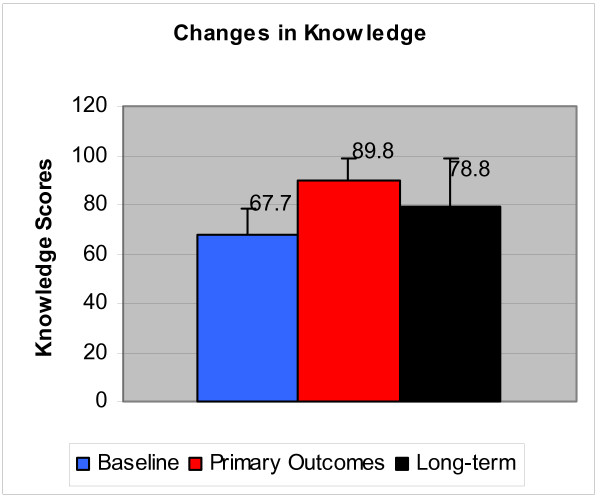
Changes in Knowledge, Confidence, and Communication. • Baseline to Long-term Outcomes P-value < 0.001 (determined by Wilcoxon). • Primary Outcome to Long-term Outcomes P-value < 0.001 (determined by Wilcoxon).

In repeated measures analyses, there were significant, sustained improvements in knowledge, confidence, and communication practices compared to baseline among those who completed questionnaires six to ten months after the course (Figure 1). Knowledge scores were highest immediately after the course, but the mean score of 78% at long-term follow-up remained higher and better than the baseline mean of 67% (P < 0.001). Confidence and communication scores continued to increase from immediately after the course to the long-term follow-up (Figure 1).

Changes in knowledge, confidence, and communications practices at the 6 to 10-month follow-up did not differ by curriculum delivery strategy. Nor were there significant differences in improvement by age, gender, profession, baseline HDS use, or having paid for CE/CME credit (data not shown). Improvements in knowledge and confidence were affected by enrollment period (fall vs spring) and whether the participant was a trainee or was in practice (Table 2). Communication practices were affected only by practice status, with trainees demonstrating greater improvements than practitioners.

## Discussion

In this long-term follow-up study, the on-line curriculum resulted in significant and sustained improvements in knowledge, confidence, and communication for diverse clinicians regardless of delivery strategy. Outcomes were only related to semester of enrollment and being a trainee versus a practitioner. Those who took the course in the spring had significantly greater improvements in knowledge and confidence scores than those enrolled in the fall. The differences between fall and spring may be because fall completers had substantially more time to forget learned information than their spring counterparts.

Similarly, trainees had significantly greater improvements than practitioners in all three outcomes (knowledge, confidence, and communication). These differences may be due to two factors. First, trainees had lower baseline scores than practitioners, allowing for greater opportunity for improvement. Secondly, trainees presumably have fewer experiences and habits to unlearn than practitioners.

As expected, knowledge scores decreased from initial follow-up to the long-term follow-up. However, even six to ten months after completing the course, knowledge scores were significantly higher than the baseline scores. This suggests significant knowledge retention of the curriculum material. Confidence and communication scores progressively increased from baseline to the long-term follow-up. These observations are consistent with the hypothesis that as individuals had more opportunity to practice the material they had learned, they could reinforce it and feel increasingly more confident and communicate with patients more comfortably.

The results of this study are consistent with previous research that demonstrate the effectiveness of online CME courses [6,7,9]. Although changes in communication in this study were statistically significant, the actual improvements were small. This is consistent with previous research which suggests low communication with patients regarding HDS use [11]. Although previous research has indicated that clinicians' behavior can be improved following training courses [8,12,13], the results of this study indicate that these behavior changes continue to improve long-term. However, additional strategies still need to be developed to more effectively improve clinician's communication practices.

This long-term follow-up study has several limitations. First, the sample consisted of self selected enrollees who elected to learn more about HDS, which limits generalizability to elective courses; it is possible that outcomes would differ for participants in required courses. Another limitation is the low response rate to the long-term follow-up. This limits the generalizability of the outcomes to those individuals who have a greater willingness to complete surveys even after the completion of the initially planned study. Those who are willing to complete such voluntary questionnaires (which were not part of the original study "contract") may have been more knowledgeable and confident about their ability to do well. This conjecture is supported by the observation that those who completed the long-term follow-up had slightly, but significantly higher knowledge scores than the non-respondents. Also, the study relied on self-reported changes in confidence and communication, which may overestimate actual behavioral changes [14, 15, 16]; future studies in this field should corroborate self-report with objective measures of clinician behavior. Finally, we did not collect information on the actual costs of delivering the curriculum through each method because study personnel were engaged in both offering and studying the intervention and did not separately allocate research and education efforts. However, it is our impression that bolus-pull delivery is the least expensive to deliver for participants such as those in this study.

## Conclusion

Despite these limitations, results from this long-term follow-up study have important implications for professional education and future research. Online case-based curriculum with evidence-based resource links results in significant and sustained improvements in knowledge, confidence, and communication. These improvements are substantial and do not appear to depend on the delivery strategy, at least among motivated clinicians. Therefore, educators can choose to offer on-line Continuing Education (CE) courses with confidence. Because the delivery strategy of online curriculum does not affect attainment of learning goals, the most convenient and low-cost delivery method can be utilized. Future studies about one-line CME should focus on whether required curriculum would have similar outcomes as elective courses as well as developing interventions that would further improve clinicians' communication patterns.

## Competing interests

The author(s) declare that they have no competing interests.

## Authors' contributions

TB drafted the manuscript.

KK conceived of the project, designed the survey and revised the manuscript.

PG reviewed and edited survey questions and revised the manuscript.

CW edited survey questions, analyzed the data and revised the manuscript.

## Pre-publication history

The pre-publication history for this paper can be accessed here:



## Figures and Tables

**Figure 2:**
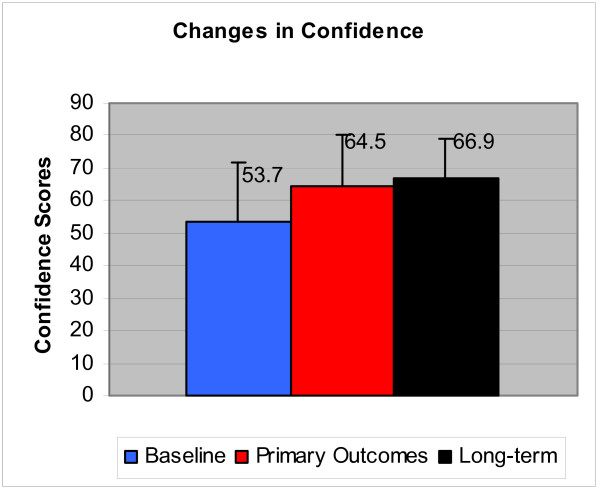
Changes in Confidence. • Baseline to Long-term Outcomes P-value < 0.001 (determined by Wilcoxon). • Primary Outcomes to Long-term Outcomes P-value Non-significant.

**Figure 3:**
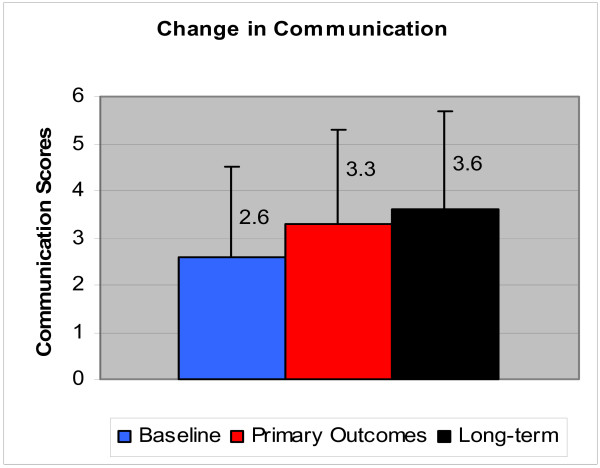
Changes in Communication. • Baseline to Long-term Outcomes P-value < 0.001 (determined by Wilcoxon). • Primary Outcomes to Long-term Outcomes P-value = 0.007 (determined by Wilcoxon).

**Table 1:** Baseline Characteristics of Non-Completers and Completers of Long-term Follow-up Questionnaire

**Characteristics**	**Non Completers**	**Completers**	**P-value**
N	395 (50.6%)	385 (49.4%)	
**Gender**			.08‡
M	101 (56.7)	77 (43.3)	
F	294 (48.8)	308 (51.2)	
**Delivery Method**			.69^
Push-bolus	93 (47.7)	102 (52.3)	
Push-drip	102 (49.5)	104 (50.5)	
Pull-bolus	93 (52.5)	84 (47.5)	
Pull-drip	107 (53.0)	95 (47.0)	
**Enrollment**			.56‡
Fall	111 (52.6)	100 (47.4)	
Spring	284 (49.9)	285 (50.1)	
**Profession**			.26^
Dietitian/RD	47 (42.0)	65 (58.0)	
Nurse/NP	111 (52.9)	99 (47.1)	
Pharmacist	25 (56.8)	19 (43.2)	
Physician/PA	115 (49.4)	118 (50.6)	
Student	97 (53.6)	84 (46.4)	
**Practice Status**			.86‡
Practitioners	262 (51.0)	252 (49.0)	
Trainees	133 (50.0)	133 (50.0)	
**Age Years (mean)**	41.8 ± 12.5	42.5 ± 12.7	.47*
**Age**			.71^
<30	104 (52.0)	96 (48.0)	
31–40	65 (53.3)	57 (46.7)	
41–50	120 (51.1)	115 (48.9)	
>50	106 (47.5)	117 (52.5)	
**Herb Use (at baseline)**			.60^
0	61 (55.0)	50 (45.0)	
1–3	128 (52.0)	118 (48.0)	
4–8	117 (47.8)	128 (52.2)	
>8	89 (50.0)	89 (50.0)	
**Baseline Total HDS Use**	5.8 ± 6.4	5.6 ± 5.3	.57†
**Course Fee Paid**			.62‡
Yes	226 (49.8)	228 (50.2)	
No	169 (51.8)	157 (48.2)	
**Baseline Knowledge (% correct)**	66.2 ± 10.8	67.7 ± 10.3	.039*
**Baseline Confidence score**	53.6 ± 18.3	53.7 ± 17.8	.92*
**Baseline Communication Practices **|	2.2 ± 2.0	2.2 ± 1.9	.44†

Confidence scale scores could range from 19, 95, with Cronbach's alpha = 0.96;

Communication scale scores could range from 0 to 10 with Cronbach's alpha = 0.84

* Determined by t-test

† Determined by Man Whitney U test

^ Determined by Pearsons Chi Square test

‡ Determined by Chi square test with continuity corrections for 2 × 2 tables

| This measure required the participant to have seen patients in the preceding 30 days

**Table 2:** Factors Associated with Changes in Expertise by Univariate Analysis

**Characteristics**	**Baseline to Long-term Follow-Up CHANGES IN Knowledge Scores**	P-value*	**Baseline to Long-term Follow-Up CHANGES IN CONF Scores**	P-value*	Baseline to Long-term Follow-Up CHANGES IN COMM Scores**	P-value*
N	385		385		182	
**Enrollment**		<.001		<.001		.48
Fall	5.4 ± 13.5		0.4 ± 25.9		1.2 ± 1.7	
Spring	11.7 ± 12.0		18.0 ± 12.9		1.0 ± 1.7	
**Practice Status**		.02		.05		.02
Practitioners	8.9 ± 12.7		11.8 ± 20.2		.9 ± 1.7	
Trainees	12.2 ± 12.5		15.9 ± 16.3		1.6 ± 1.6	

• The p-values were calculated using Mann-Whitney U tests.

• ** The number of subjects answering the questions about communication practices was lower because these questions were only answered by participants who reported having seen a patient in the 30 days prior to the survey baseline, initial follow-up, and long-term follow-up.

## References

[B1] Izzo AA (2005). Herb-drug interactions: an overview of the clinical evidence. Fundam Clin Pharmacol.

[B2] Kemper KJ, Amata-Kynvi A, Dvorkin L, Whelan JS, Woolf A, Samuels RC, Hibberd P (2003). Herbs and other dietary supplements: healthcare professionals' knowledge, attitudes, and practices. Altern Ther Health Med.

[B3] Kreitzer MJ, Mitten D, Harris I, Shandeling J (2002). Attitudes toward CAM among medical, nursing, and pharmacy faculty and students: a comparative analysis. Altern Ther Health Med.

[B4] Whitcomb ME (2002). CME reform: an imperative for improving the quality of medical care. Acad Med.

[B5] Fordis M, King JE, Ballantyne CM, Jones PH, Schneider KH, Spann SJ, Greenberg SB, Greisinger AJ (2005). Comparison of the instructional efficacy of Internet-based CME with live interactive CME workshops: a randomized controlled trial. JAMA.

[B6] Davis D, O'Brien MA, Freemantle N, Wolf FM, Mazmanian P, Taylor-Vaisey A (1999). Impact of formal continuing medical education: do conferences, workshops, rounds, and other traditional continuing education activities change physician behavior or health care outcomes?. JAMA.

[B7] Kemper KJ, Amata-Kynvi A, Sanghavi D, Whelan JS, Dvorkin L, Woolf A, Samuels RC, Hibberd P (2002). Randomized trial of an internet curriculum on herbs and other dietary supplements for health care professionals. Acad Med.

[B8] Kemper KJ, Gardiner P, Gobble J, Mitra A, Woods C (2006). Randomized Controlled Trial Comparing Four Strategies for Delivering e-Curriculum to Health Care Professionals [ISRCTN88148532]. BMC Med Educ.

[B9] Kemper KJ, Gardiner P, Gobble J, Woods C (2006). Expertise about herbs and dietary supplements among diverse health professionals. BMC Complement Altern Med.

[B10] Jaski ME, Schwartzberg JG, Guttman RA, Noorani M (2000). Medication review and documentation in physician office practice. Eff Clin Pract.

[B11] Cockayne NL, Duguid M, Shenfield GM (2005). Health professionals rarely record history of complementary and alternative medicines. Br J Clin Pharmacol.

[B12] Hudson K, Brady E, Rapp D (2001). What you and your patients should know about herbal medicines. JAAPA.

[B13] Fowles JB, Rosheim K, Fowler EJ, Craft C, Arrichiello L (1999). The validity of self-reported diabetes quality of care measures. Int J Qual Health Care.

[B14] Glintborg B, Andersen SE, Spang-Hanssen E, Dalhoff K (2005). Disregarded use of herbal medical products and dietary supplements among surgical and medical patients as estimated by home inspection and interview. Pharmacoepidemiol Drug Saf.

[B15] Veninga CC, Denig P, Pont LG, Haaijer-Ruskamp FM (2001). Comparison of indicators assessing the quality of drug prescribing for asthma. Health Serv Res.

